# Interoceptive fear learning to mild breathlessness as a laboratory model for unexpected panic attacks

**DOI:** 10.3389/fpsyg.2015.01150

**Published:** 2015-08-06

**Authors:** Meike Pappens, Evelien Vandenbossche, Omer Van den Bergh, Ilse Van Diest

**Affiliations:** Health Psychology, University of LeuvenLeuven, Belgium

**Keywords:** fear conditioning, panic, respiration, interoceptive fear, CS–US contingency, dual process theory

## Abstract

Fear learning is thought to play an important role in panic disorder. Benign interoceptive sensations can become predictors (conditioned stimuli – CSs) of massive fear when experienced in the context of an initial panic attack (unconditioned stimulus – US). The mere encounter of these CSs on a later moment can induce anxiety and fear, and precipitate a new panic attack. It has been suggested that fear learning to interoceptive cues would result in unpredictable panic. The present study aimed to investigate whether fear learning to an interoceptive CS is possible without declarative knowledge of the CS–US contingency. The CS consisted of mild breathlessness (or: dyspnea), the US was a suffocation experience. During acquisition, the experimental group received six presentations of mild breathlessness immediately followed by suffocation; for the control group both experiences were always separated by an intertrial interval. In the subsequent extinction phase, participants received six unreinforced presentations of the CS. Expectancy of the US was rated continuously and startle eyeblink electromyographic, skin conductance, and respiration were measured. Declarative knowledge of the CS–US relationship was also assessed with a post-experimental questionnaire. At the end of acquisition, both groups displayed the same levels of US expectancy and skin conductance in response to the CS, but the experimental group showed a fear potentiated startle eyeblink and a different respiratory response to the CS compared to the control group. Further analyses on a subgroup of CS–US unaware participants confirmed the presence of startle eyeblink conditioning in the experimental group but not in the control group. Our findings suggest that interoceptive fear learning is not dependent on declarative knowledge of the CS–US relationship. The present interoceptive fear conditioning paradigm may serve as an ecologically valid laboratory model for unexpected panic attacks.

## Introduction

Fear learning to initially harmless cues is considered one of the mechanisms in the etiology of panic disorder (Bouton et al., 2001; Kessler et al., 2006; Acheson et al., 2012; De Cort et al., 2012; Pappens et al., 2012). Low-level interoceptive bodily sensations (such as mild sensations of dyspnea; a slight increase in heart rate; etc.) are thought to acquire fear-evoking properties because they were once experienced in the context of an isolated panic attack (unconditioned stimulus – US). A panic-predictive connotation is attributed to the cues (conditioned stimuli – CSs), causing them to trigger anxiety and/or fear on a later encounter. The successful application of exposure techniques in the treatment of panic disorder demonstrates the relevance of learning theory for this disorder. For example, interoceptive exposure to mild bodily sensations significantly reduces long-term anxiety in panic patients (McHugh et al., 2009).

An important feature of panic attacks is that they can occur either ‘expectedly’ or ‘unexpectedly’ (DSM-V). It has been suggested that especially in the latter case fear conditioning to an interoceptive sensation is responsible for triggering a new culmination of fear and fear-related arousal (Vickers and McNally, 2005; Johnson et al., 2014).

Up until today interoceptive fear conditioning remains a poorly studied phenomenon, despite the fact that approximately 40% of panic attacks are defined as unexpected (Shulman et al., 1994). Over the past years, a few studies have investigated fear conditioning to an interoceptive CS in the laboratory. For example, Acheson et al. (2007) used 5 s applications of 20% CO_2_-enriched air as an interoceptive CS and 15 s of the same substance as US in a panic-relevant interoceptive fear conditioning design. In a follow-up study (Acheson et al., 2012) they investigated discriminatory learning in interoceptive fear conditioning. Earlier, we successfully established and extinguished fear to a mild sensation of breathlessness by combining it with a distressing breathing obstruction and investigated the differences between exteroceptive versus interoceptive fear conditioning and the influence of vagal tone on interoceptive fear conditioning processes (Pappens et al., 2012, 2013, 2014). Together these studies demonstrated that fear can be acquired to benign interoceptive sensations and they provided valid experimental paradigms to study the characteristics of interoceptive fear conditioning in the laboratory.

An unstudied yet fundamental issue remains whether interoceptive fear conditioning can happen outside of awareness as is suggested by its presumable role in unpredictable panic. For decades, there has been an ongoing discussion in the scientific community on the necessity of propositional knowledge of the CS–US contingency as a prerequisite for associative fear learning. Proponents of a single-process theory of fear learning consider high-level cognitive processes as the foundation of all associative learning. As a consequence fear learning is based on propositional knowledge of the CS–US contingency (e.g., Purkis and Lipp, 2001; Lovibond and Shanks, 2002; Mitchell et al., 2009; Sebastiani et al., 2011; Newell and Shanks, 2014).

In contrast, a dual-process theory of fear learning describes one type of learning that leads to declarative knowledge and another type that activates the fear system without the person being necessarily aware of the CS–US contingency (e.g., Hamm and Vaitl, 1996; LeDoux, 1996; Clark and Squire, 1998; Öhman and Soares, 1998; Öhman and Mineka, 2001; Knight et al., 2003; Beaver et al., 2005; Smith et al., 2005; Tabbert et al., 2006, 2011; Weike et al., 2007; Kindt et al., 2009; Raes et al., 2010; Schultz and Helmstetter, 2010; Soeter and Kindt, 2010). In the past years, a great number of studies have sought to provide evidence for the existence of two distinct fear learning pathways. For example, Beaver et al. (2005) demonstrated fear learning to a subliminally administered – and thus not consciously perceived- danger cue (CS+). Also, fear learning to a CS+ was present in skin conductance responses (SCRs) although it was hardly discriminable from a CS- (Schultz and Helmstetter, 2010), but these results could not be replicated (Sevenster et al., 2014). Clark and Squire (1998) examined startle eyeblink responses in a classical aversive conditioning study in patients with deficits in declarative memory as a result of severe bilateral hippocampal lesions. As expected, the patients were not capable of reporting upon the CS–US relationship. However, they did display fear potentiated startle eyeblink responses to the danger cue, which were probably triggered through activation of the amygdala (LeDoux, 2000). Also, the intake of propranolol, a substance blocking noradrenergic release (Kindt et al., 2009) resulted in a dissociation in fear learning outcomes (self-reported fear and the startle eyeblink response on the one hand, versus skin conductance and US-expectancy ratings on the other hand).

In a previous study in healthy participants we also demonstrated a divergence between different fear measures in a panic-relevant interoceptive fear conditioning paradigm (Pappens et al., 2013). The experimental group received six paired administrations of a CS (a mild feeling of breathlessness) and a US (a strong feeling of breathlessness) during acquisition. The control group received the same amount of CSs and USs in acquisition, but in an unpaired fashion. Extinction was the same for both groups – it consisted of six CS-only administrations. Our measures included startle eyeblink responses electromyographic (EMG), respiratory measures and self-reported fear. While fear conditioning emerged in startle eyeblink EMG and respiration in the experimental group only, no group differences occurred in self-reported fear. Thus, although the experimental group showed a potentiated startle eyeblink response and an increased ventilation during the CS, they did not to report more fear to the CS compared to the control group. These findings suggest that our interoceptive paradigm is able to install fear without propositional knowledge of its presence. However, a direct measure of declarative knowledge about the CS–US contingency was lacking in the study nor was it possible to compare participants that were either aware or not of the CS–US relationship. This limited the conclusions that could be drawn with respect to the necessity of propositional knowledge to install interoceptive fear learning or existence of fear learning.

With the current study we aimed to replicate and extend our previous study. We replaced self-reported fear by a continuous measure of self-reported US-expectancy (DIAL) and we included SCRs. Declarative knowledge of the CS–US relationship was also evaluated post-experimentally by assessing the participants’ knowledge of the experienced contingencies.

Our hypotheses were the following:

We expected to replicate the findings of Pappens et al. (2013) during acquisition: (a) higher startle eyeblink EMG responses during the CS than during ITI in the experimental but not in the control group; (b) less reduction in respiratory frequency and in tidal volume during the CS load in the experimental compared to the control group; and we predicted: (c) US expectancy during the CS load not to be greater for the experimental than for the control group. (d) since SCR was not included in Pappens et al. (2013) and given the mixed results in literature (e.g., Kindt et al., 2009; Schultz and Helmstetter, 2010; Sevenster et al., 2014) we held no specific predictions for this outcome measure.

Given the hypothesized role of interoceptive fear learning in unexpected panic, we expected to observe similar differences during acquisition in the experimental subgroup of CS–US unaware participants versus those in the control group: (a) higher startle eyeblink EMG responses during the CS than during ITI in the experimental but not in the control group; (b) less of a reduction in respiratory frequency and in tidal volume during the CS load in the experimental compared to the control group; (c) no differences in US expectancy in the experimental versus control group; (d) again, we held no specific predictions for SCR.

## Materials and Methods

### Participants

Fifty-six psychology students participated in this study (51 women, *M* = 19.16 years, range 18–25 years) in return for a course credit. Persons with a current or past history of cardiovascular disease, chronic, or acute respiratory disease, pregnancy, current, or past history of drug or alcohol abuse or dependence, psychotropic drug use and any current or past psychiatric disorder including panic and anxiety disorder were excluded from the study. The study protocol was approved by the Medical Ethics Committee of the University of Leuven in accordance with the Declaration of Helsinki. All subjects signed an informed consent form stating – amongst other information – that participation was voluntary and that they could withdraw from the study at any moment.

### Stimuli and Apparatus

#### Stimuli

The CS consisted of a non-aversive linear resistive load of 0.98 kPa^∗^s/l, whereas an aversive linear resistive load of 3.91 kPa^∗^s/l served as the US. Resistive loads were administered during both the inspiratory and expiratory phases of the respiratory cycle. The CS was presented for 8 s, the US for 30 s (Pappens et al., 2013).

#### Breathing Apparatus

A mouthpiece was mounted onto a bacterial filter that was fitted on a pneumotachograph (Fleisch No. 2, Epalinges, Switzerland). The pneumotachograph was connected to a non-rebreathing valve of which the inspiratory and expiratory port were installed on a three-way *Y*-valve (stopcock type) using a vinyl tube (inner diameter: 3.5 cm; length 100 cm). This set-up enabled easy switching between room air and loaded breathing. The signal from the pneumotachograph was amplified using a pressure transducer (Sine Wave Carrier Demodulator CD15, Valydine EngineeringTM) and was calibrated daily with a 1 l syringe. Fractional end-tidal CO_2_ (FetCO_2_) was measured using an infrared capnograph (POET II, Criticare, USA) that sampled expired air from the breathing circuit close to the mouthpiece. The capnograph was calibrated daily using a calibration gas containing 7.5% CO_2_. Air flow and CO_2_ waveforms were digitized at 20 Hz.

#### Skin Conductance

Electrodermal activity was recorded with Fukuda standard Ag/AgCl electrodes (1 cm diameter) filled with a Unibase electrolyte and attached to the hypothenar palm of the non-dominant hand, which was cleaned with tap water before the start of the procedure. The inter-electrode distance was 2.5 cm. A Coulbourn skin conductance coupler (LabLinc v71–23) provided a constant 0.5 V across electrodes. The signal was digitized at 100 Hz.

#### Startle Eyeblink Response

Orbicularis oculi electromyographic activity (EMG) was recorded as an index of the eyeblink component of the startle response with three Ag/AgCl Sensormedics electrodes (0.25 cm diameter) filled with electrolyte gel. After cleaning the skin to reduce inter-electrode resistance, electrodes were placed on the left side of the face (Blumenthal et al., 2005). The raw signal was amplified by a Coulbourn isolated bioamplifier with bandpass filter (LabLinc v75–04). The recording bandwidth of the EMG signal was between 13 Hz and 10 kHz. The signal was rectified online and smoothed by a Coulbourn multifunction integrator (LabLinc v76–23 A) with a time constant of 50 ms. The EMG signal was digitized at 1000 Hz from 500 ms before the onset of the auditory startle probe (a 95 dB burst of white noise with a rise time <1 ms presented binaurally for 50 ms through headphones) until 1000 ms after probe onset.

#### US Expectancy Dial

Participants were asked to continuously rate their expectancy of the US with a custom built dial (Pappens et al., 2012). The scale on which they had to rate US-expectancy, ranged from zero (“I am certain that the heavy breathing resistance is not coming now”) to one hundred (“I am certain that the heavy breathing resistance is coming now”). The dial produced an analog signal, which was digitized and stored at 10 Hz (Pappens et al., 2012).

#### Post-Experimental Contingency Assessment

After the experimental phase, participants were questioned orally about their knowledge of the CS–US relationship with the following open question: “can you tell us whether you detected a certain order in the administration of the different types of stimuli?” Participants of the experimental group were categorized as ‘aware’ when correctly identifying that a light breathing resistance (CS) immediately preceded a strong breathing resistance (US) and as ‘unaware’ if they could not verbalize this relationship. In the control group a person was labeled as ‘aware’ when he/she correctly identified that CS and US were separated by an intertrial interval (ITI) and as ‘unaware’ if they did not.

#### Software

All devices were connected to a PC through a National Instruments PCI-6221 16-Bit acquisition card (National Instruments, Austin, TX, USA). Affect 4.0 software (Spruyt et al., 2010) was used for stimulus presentation and data acquisition. Physiological signals were treated off-line with PSPHA (De Clerck et al., 2006), a modular script-based program to generate and apply calibration factors and to extract parameters from each of the recorded signals.

### Procedure

Upon their arrival, the experimenter told the participants that psychophysiological and subjective responses would be measured during three different types of breathing trials: normal breathing, mildly restricted breathing and heavily restricted breathing. She showed the participants how to continuously indicate their US expectancy with the online dial. Then she attached the electrodes and explained that brief bursts of noise would be administered through the headphones but that these could be ignored. Following this, participants took in the mouthpiece and put on the nose-clip and the headphones.

After the administration of 10 acoustic startle probes (30 s in between probes) to habituate the startle eyeblink response the experimental phase was started. Participants received three CS pre-exposure trials, followed by six acquisition trials and six extinction trials. Half of the participants were randomly assigned to the experimental (paired) group, the other half to the control (unpaired) group.

A pre-exposure trial consisted of a 20 s baseline, a CS (8 s) presentation and an ITI of 30 s without stimulus. For the experimental group, acquisition trials consisted of baseline (20 s), CS (8 s), US (30 s), and ITI (variable between 25 and 35 s). The control group received the following sequence during the acquisition trials: baseline (20 s), CS (8 s), ITI (25–35 s), and US (30 s). Extinction trials consisted of baseline (20 s), CS (8 s), and ITI (55–65 s) for both groups. Startle probes were applied in every trial at random times between 6.5–7.5 s after CS onset, between 21–23 s after US onset, and between 21–23 s after ITI onset (Pappens et al., 2013). After the experimental phase of the study participants were questioned about their knowledge of the CS–US relationship.

Before leaving, participants were fully debriefed.

### Response Definition, Scoring, and Statistical Analysis

Startle eyeblink EMG data, SCRs and respiratory signals were processed with PSychoPHysiological Analysis (PSPHA; De Clerck et al., 2006).

The EMG response was calculated by subtracting the mean baseline value (0–20 ms after probe onset) from the peak value (between 21–175 ms after probe onset). Each startle eyeblink was visually checked on artifacts. Distorted blinks or trials with spontaneous blinks during baseline were removed from the data. Rejected blinks (*n* = 11) were replaced by the means of the previous and following trial. EMG data were standardized and T-transformed within persons (Blumenthal et al., 2005).

Respiratory rate (RR, in cycles per minute, cpm) and tidal volume (V_T_, in ml) were extracted on a breath-by-breath basis and then averaged across the CS episode, and across the 20 s baseline episode preceding the CS. Baseline values were subtracted from CS values for statistical analysis.

Skin conductance responses were calculated by subtracting the mean skin conductance level (SCL) during baseline (2 s before the CS onset) from the maximum SCR during the subsequent 8 s CS period. All SCRs below 0.05 were coded as a non-response by setting them to zero and values were Log10 (SCR + 1) transformed.

The mean of the online US-expectancy ratings (DIAL) were calculated for the first and the last second of the CS presentation per trial.

All data (EMG, RR, V_T_, SCR, DIAL) were averaged across three pre-exposure trials and across every two subsequent trials in acquisition and extinction, resulting in one pre-exposure block, three acquisition blocks, and three extinction blocks.

Due to technical difficulties, the data of five participants (two of the experimental group and three of the control group) were excluded from all analyses, bringing the total number of participants on 51. For DIAL and respiration, data of one additional participant of the experimental group was lost due to technical difficulties.

Potential pre-existing group differences were tested with a first set of analyses on the pre-exposure data. Respiratory parameters and SCR data were entered in a repeated measures ANOVA (RM ANOVA) design with group (experimental/control) as a between subject variable. Probe (CS/ITI) and Time (second 1, second 8) were, respectively, added as within subject variables for startle eyeblink EMG and DIAL.

The *a priori* hypotheses that served to examine whether we could replicate the results of Pappens et al. (2013) were analyzed in mixed ANOVA designs. Startle eyeblink EMG data were entered in a group (experimental/control) × probe (CS/ITI) × block (1, 2, 3) design. Respiratory parameters and SCR data were entered in a design with group (experimental/control) and block (1, 2, 3) as a between subject and a within subject variable respectively. DIAL data were entered in a group (experimental/control) × time (second 1, second 8) × block (1, 2, 3) design. Only acquisition data were entered in the analyses – extinction data are depicted in the figures for illustrative purposes only.

Participants were assigned to the ‘aware’ or ‘unaware’ group based on the post-experimental assessment of awareness. We opted to use this CS–US contingency awareness measure and not the CS–US expectancy dial because it produced a discrete ‘YES’ or ‘NO’ awareness criterion. (Data of the CS–US expectancy dial are more difficult to unambiguously categorize as aware/not aware in a between-subject design and less clearly result in a dichotomy. Former studies who did use online dial data for categorization were within-subject paradigms; e.g., Schultz and Helmstetter, 2010).

Using the post-experimental CS–US contingency awareness data, 35 of 51 included participants were labeled as ‘unaware’; 16 in the experimental group and 19 in the control group. For DIAL and respiration, 34 ‘unaware’ and 16 ‘aware’ persons were identified in the 50 included participants.

Hypotheses were tested also in the ‘unaware’ subgroup by entering the data in a mixed ANOVA design with group (experimental/control) as a between-subject variable and block (1, 2, 3) as a within-subject variable. Probe (CS/ITI) and Time (Second 1, Second 8) were added as a within-subject variable for the EMG and US-expectancy analyses, respectively.

Alpha was set at 0.05. Greenhouse–Geisser corrections were applied where appropriate. Uncorrected degrees of freedom and corrected *p*s are reported, together with $\eta_{p}^{2}$. Additional testing of significant results were analyzed with two-tailed planned comparisons. Statistical analyses for all measures were accomplished with Statistical Version 12.

## Results

### Pre-Exposure

#### US-Expectancy

No effects involving the group factor were observed in US expectation. Main effect of group: *F*(1,48) = 0.54, *p* = 0.47, $\eta_{p}^{2}$ = 0.01; group × time: *F*(1,48) = 0.54, *p* = 0.47, $\eta_{p}^{2}$ = 0.01.

**FIGURE 1:**
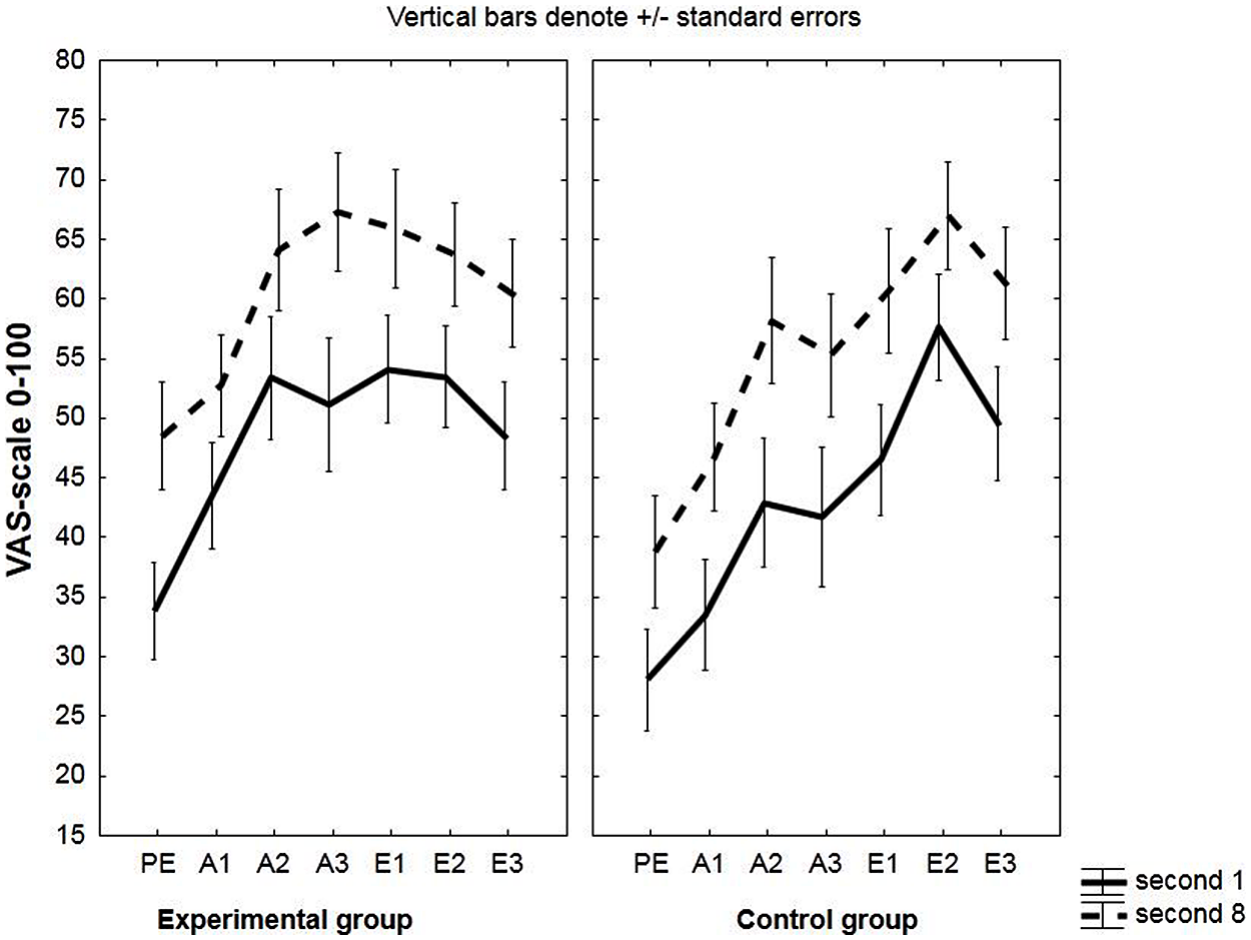
**Unconditioned stimulus (US)-expectancy during the conditioned stimulus (CS)-period.** Mean US-expectancy ratings of second 1 and second 8 during the conditioned stimuli (CSs) on a VAS-scale ranging from 0 (certainly no breathing resistance) to 100 (certainly breathing resistance) for the experimental and the control group. Responses were averaged across three pre-exposure trials (PE), two acquisition trials (A1, A2, A3) and two extinction trials (E1, E2, E3).

US-expectancy increased from second 1 to second 8; main effect of time: *F*(1,48) = 22.88, *p* < 0.001, $\eta_{p}^{2}$ = 0.32, see **Figure 1**.

#### Startle Eyeblink EMG

**FIGURE 2:**
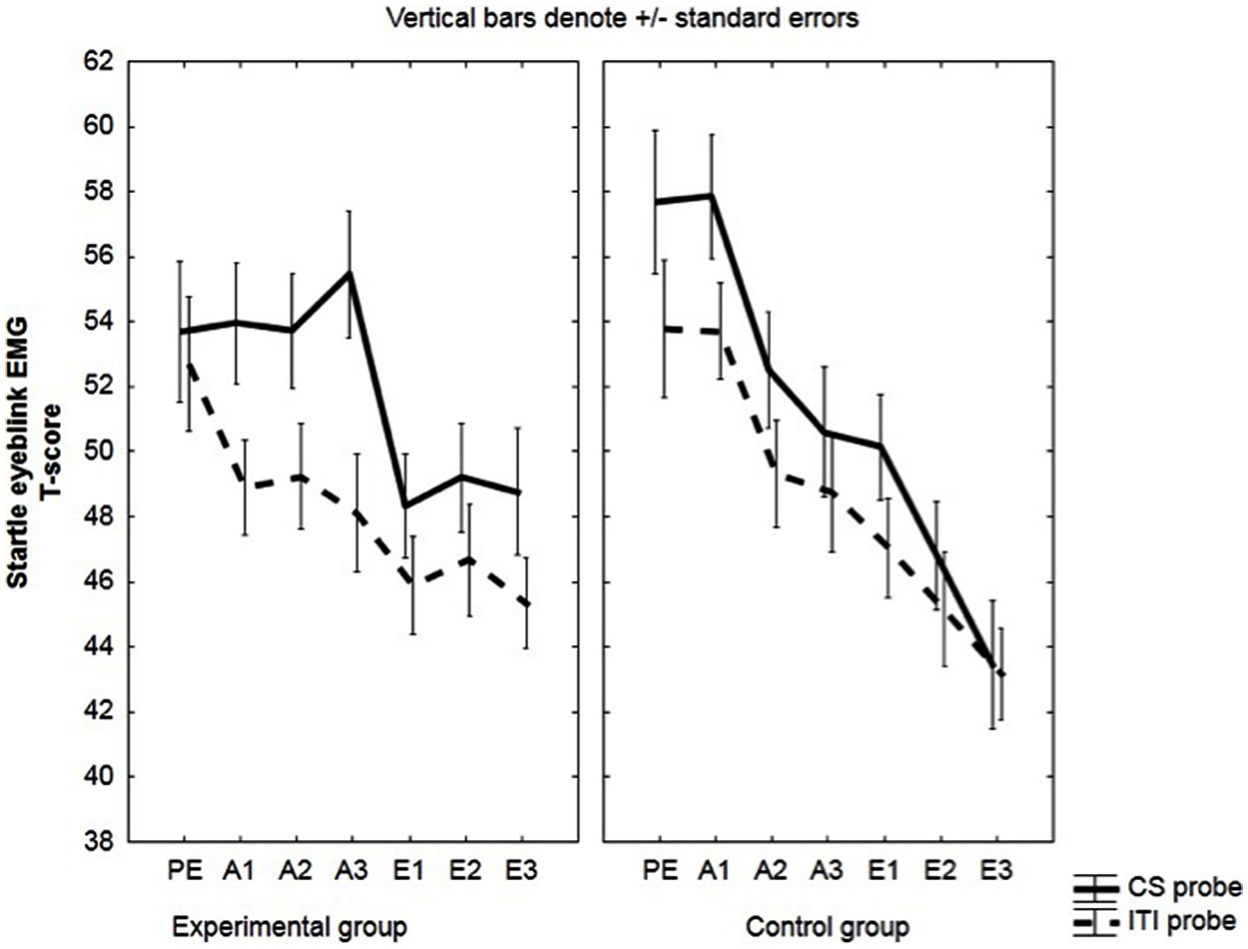
**Startle eyeblink responses electromyographic (EMG).** Mean EMG-responses (*T*-scores) during the CS and during ITI for the experimental and the control group. Startle eyeblink responses were averaged across three pre-exposure trials (PE), two acquisition trials (A1, A2, A3) and two extinction trials (E1, E2, E3).

No *a priori* group differences were found in startle eyeblink data during pre-exposure: group: *F*(1,49) = 0.94, *p* = 0.34, $\eta_{p}^{2}$ = 0.02; group × probe: *F*(1,49) = 0.88, n.s., $\eta_{p}^{2}$ = 0.02, see **Figure 2**.

#### Respiratory Rate

**FIGURE 3:**
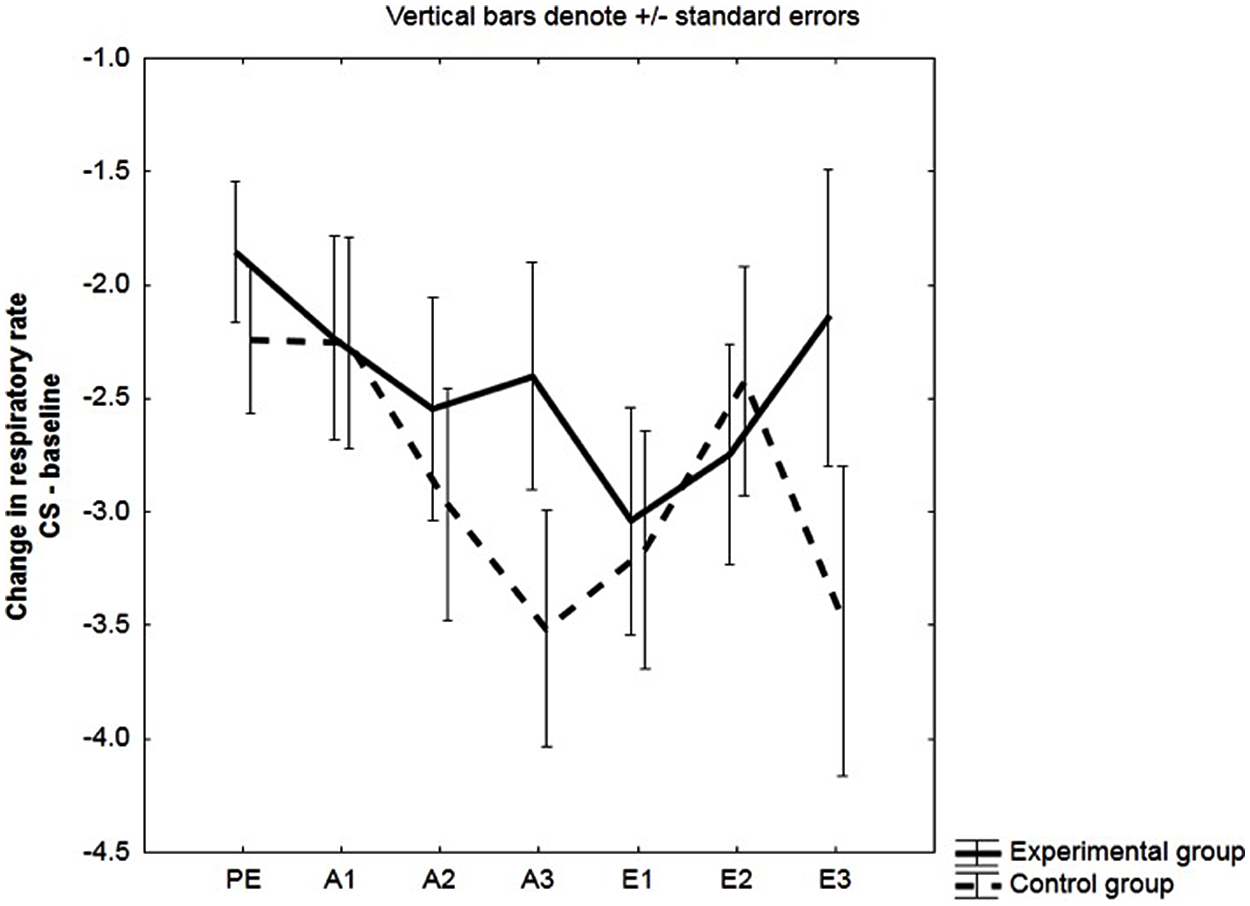
**Change in respiratory rate (RR).** Mean changes in RR (in cycles per minute, cpm) during the CS relative to baseline (CS minus baseline) for the experimental and the control group. Respiratory responses were averaged across three pre-exposure trials (PE), two acquisition trials (A1, A2, A3) and two extinction trials (E1, E2, E3).

As expected, the effect of group was not significant: *F*(1,48) = 0.74, *p* = 0.40, $\eta_{p}^{2}$ = 0.015, see **Figure 3**.

#### Tidal Volume (V_T_)

**FIGURE 4:**
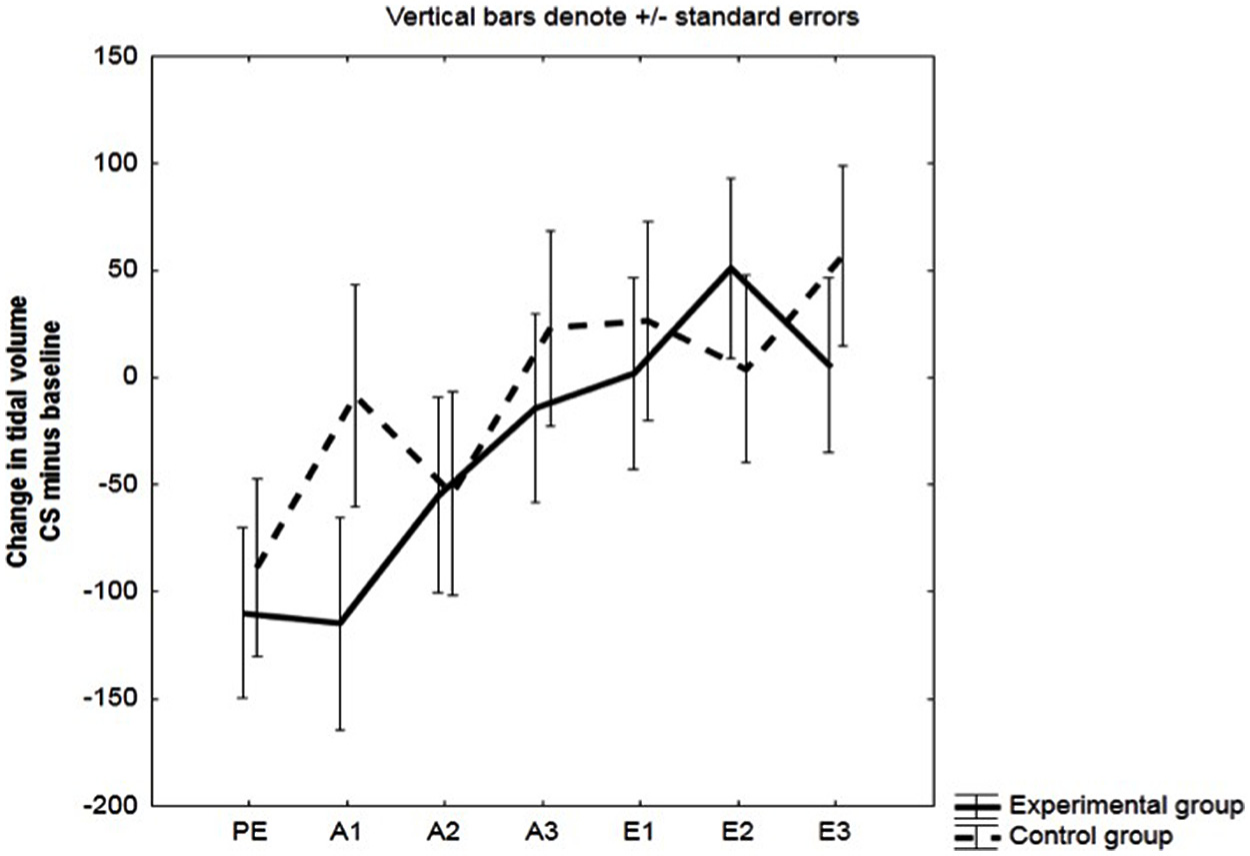
**Change in Tidal Volume (V_T_).** Mean changes in V_T_ (in ml) during the CS relative to baseline (CS minus baseline) for the experimental and the control group. Respiratory responses were averaged across three pre-exposure trials (PE), two acquisition trials (A1, A2, A3), and two extinction trials (E1, E2, E3).

We observed no differences between the experimental and the control group in V_T_ during pre-exposure: *F*(1,48) = 0.14, *p* = 0.71, $\eta_{p}^{2}$ = 0.003. See **Figure 4**.

#### Skin Conductance Response

**FIGURE 5:**
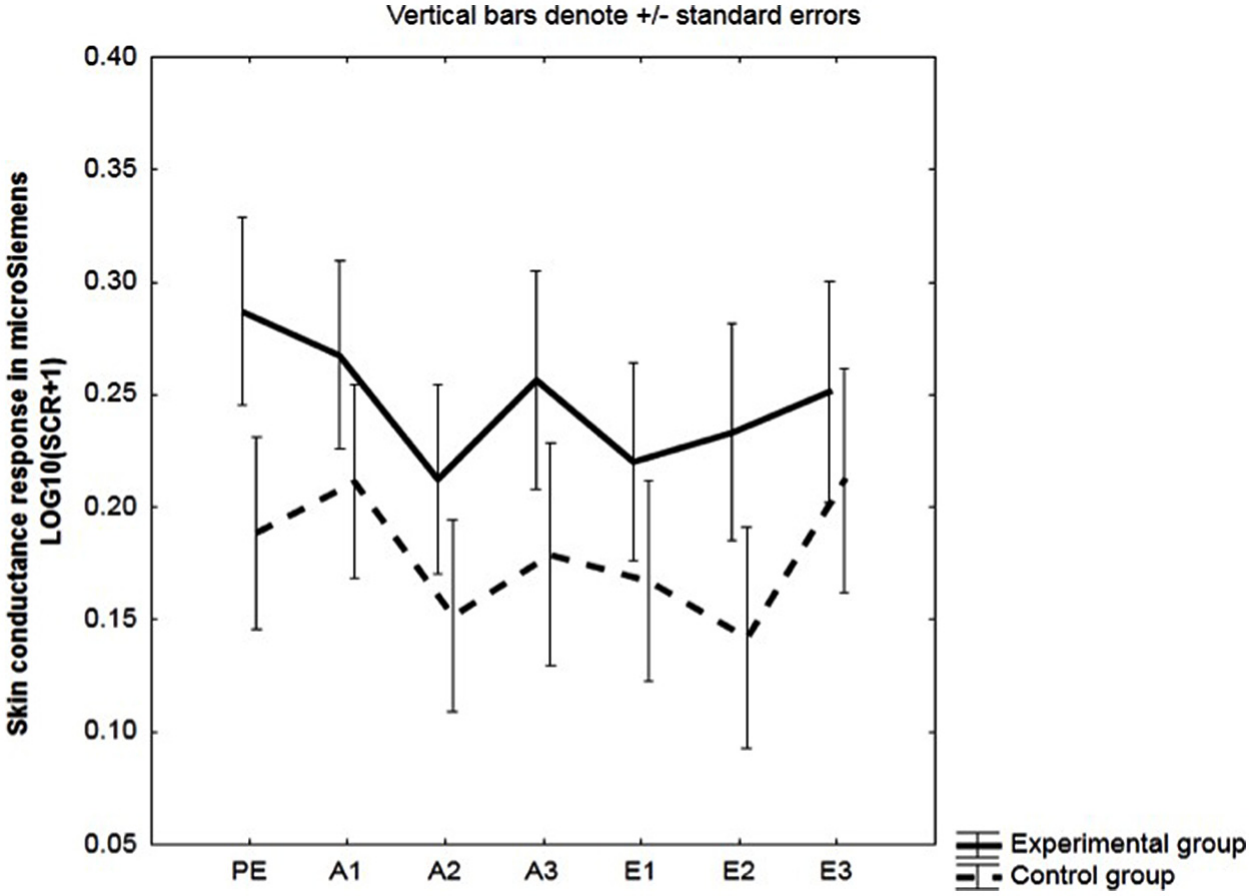
**Skin conductance responses (SCR) during the CS-period.** Mean SCRs [LOG10 (1 + SCR)] in microSiemens during the CSs. SCRs were averaged across three pre-exposure trials (PE), two acquisition trials (A1, A2, A3), and two extinction trials (E1, E2, E3).

Although **Figure 5** suggests that the experimental group displayed higher SCRs during pre-exposure, such effect of group was not significant: *F*(1,49) = 2.75, *p* = 0.10, $\eta_{p}^{2}$ = 0.05.

### Replication of Pappens et al. (2013)

#### US-Expectancy

No significant effects involving the group factor were observed in US expectancy. Effect of group: *F*(1,48) = 2.69, *p* = 0.11, $\eta_{p}^{2}$ = 0.05; group × block: *F*(2,96) = 0.17, *p* = 0.85, $\eta_{p}^{2}$ = 0.003; group × time: *F*(1,48) = 0.12, *p* = 0.73, $\eta_{p}^{2}$ = 0.003. The critical group × block × time interaction was also not significant: *F*(2,96) = 0.62, *p* = 0.54, $\eta_{p}^{2}$ = 0.01.

Participants’ US expectancy increased from the first to the last second of the CS; main effect of time: *F*(1,48) = 20.99, *p* < 001, $\eta_{p}^{2}$ = 0.30. Ratings also increased over blocks; main effect of block: *F*(2,96) = 10.24, *p* < 0.001, $\eta_{p}^{2}$ = 0.18. See **Figure 1**.

#### Startle Eyeblink EMG

Further testing of the significant probe x group interaction [*F*(1,49) = 7.99, *p* < 0.01, $\eta_{p}^{2}$ = 0.14] indicated that CS probes evoked larger responses than ITI probes in the experimental group, *F*(1,49) = 23.01, *p* < 0.001, but not in the control group, *F*(1,49) = 0.73, *p* = 0.40.

The main effect of group was not significant, *F*(1,49) = 0.01, *p* = 0.91, $\eta_{p}^{2}$ = 0.00; nor was the block × group interaction, *F*(2,98) = 1.07, *p* = 0.35, $\eta_{p}^{2}$ = 0.02, or the probe × block × group interaction, *F*(2,98) = 0.18, *p* = 0.83, $\eta_{p}^{2}$ = 0.00.

We also observed a significant effect of probe: *F*(1,49) = 16.18, *p*<0.01, $\eta_{p}^{2}$ = 0.25. See **Figure 2**.

#### Respiratory Rate

Both the main effect of group and the group × block interaction were not significant; *F*(1,48) = 0.89, *p* = 0.35, $\eta_{p}^{2}$ = 0.02, and *F*(2,96) = 1.13, *p* = 0.33, $\eta_{p}^{2}$ = 0.02, respectively.

However, in line with results of Pappens et al. (2013) and as suggested by **Figure 3**, two-tailed planned comparisons did indicate that a linear decrease in RR in response to the CS (change scores from baseline to CS) from the first to the last block of acquisition was not significant for the experimental group, *F*(1,48) = 0.10, *p* = 0.76, while it was for the control group, *F*(1,48) = 5.18, *p* = 0.013 (see **Figure 3**). This effect was mainly driven by an increase in RR in the control group during baseline (data not presented).

#### Tidal Volume (V_T_)

No significant effects involving the group factor were observed: group, *F*(1,48) = 0.58, *p* = 0.45, $\eta_{p}^{2}$ = 0.01; group × block interaction, *F*(2,96) = 2.09, *p* = 0.13, $\eta_{p}^{2}$ = 0.04. See **Figure 4**.

#### Skin Conductance Response

No significant effects involving the group factor emerged for SCR;

group: *F*(1,49) = 1.29, *p* = 0.26, $\eta_{p}^{2}$ = 0.03; group × block interaction: *F*(2,98) = 0.11, *p* = 0.90, $\eta_{p}^{2}$ = 0.002, see **Figure 5**.

### Analysis on Subgroup of ‘Unaware’ Participants

#### US Expectancy

No differences in US expectancy were observed between the experimental and the control group during acquisition. Effect of group: *F*(1,32) = 0.94, *p* = 0.34, $\eta_{p}^{2}$ = 0.03; group × time:, *F*(1,32) = 0.25, *p* = 0.62, $\eta_{p}^{2}$ = 0.008; group × block:, *F*(2,64) = 0.40, *p* = 0.67, $\eta_{p}^{2}$ = 0.01; group × block × time: *F*(2,64) = 1.27, *p* = 0.29, $\eta_{p}^{2}$ = 0.04.

A significant main effect of time was present, *F*(1,32) = 22.88, *p* < 0.001, $\eta_{p}^{2}$ = 0.42, with an increase in US expectancy during the CS from second 1 to second 8; and also of block, *F*(2,64) = 7.73, *p* < 0.001, $\eta_{p}^{2}$ = 0.19, indicating that US expectancy augmented across acquisition blocks. See **Figure 8**.

#### Startle Eyeblink EMG

Across acquisition, a decrease in startle responses during the CS probe was observed for the control group, *F*(1,33) = 17.12, *p* < 0.001, but not for the experimental group, *F*(1,33) = 0.75, *p* = 0.39; significant group × probe × block interaction: *F*(2,66) = 3.26, *p* = 0.04, $\eta_{p}^{2}$ = 0.09.

Also the two-way interactions were significant; probe × group interaction: *F*(1,33) = 4.69, *p* = 0.04, $\eta_{p}^{2}$ = 0.12, and block × group interaction: *F*(2,66) = 5.56, *p* < 0.01, $\eta_{p}^{2}$ = 0.14.

**FIGURE 6:**
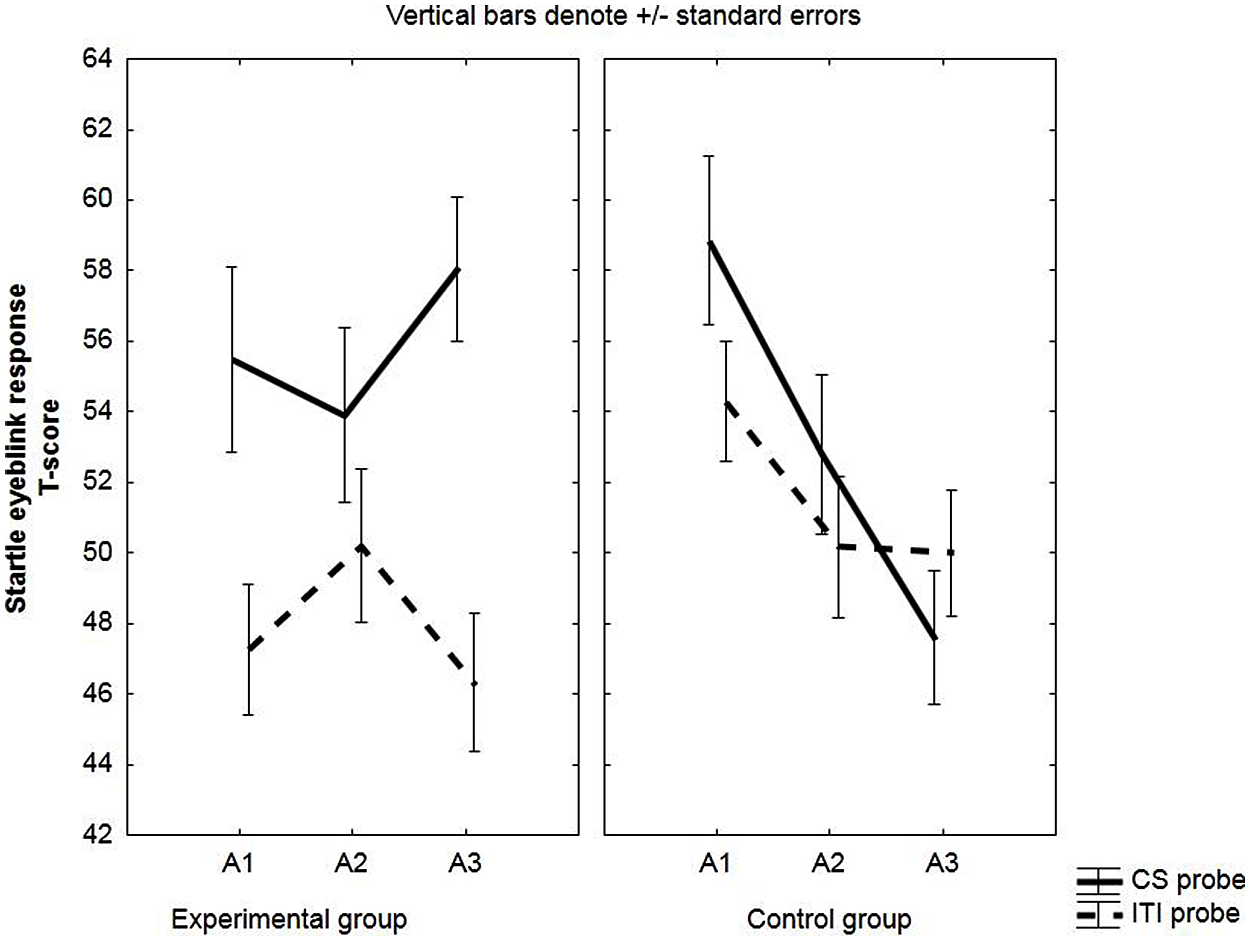
**Startle eyeblink responses in unaware participants (EMG).** Mean EMG-responses (*T*-scores) during the CS and during ITI for the experimental and the control group. Startle eyeblink responses were averaged across two acquisition trials (A1, A2, A3).

Finally, probes administered during the CS evoked stronger startle responses than those during ITI; main effect of probe: *F*(1,33) = 10.7, *p* < 0.01, $\eta_{p}^{2}$ = 0.24). See **Figure 6**.

#### Respiratory Rate

We observed no differences in RR between the experimental and the control group. Effect of Group: *F*(1,32) = 1.91, *p* = 0.18, $\eta_{p}^{2}$ = 0.06. Group × Block interaction: *F*(2,64) = 1.26, *p* = 0.29, $\eta_{p}^{2}$ = 0.04.

**FIGURE 7:**
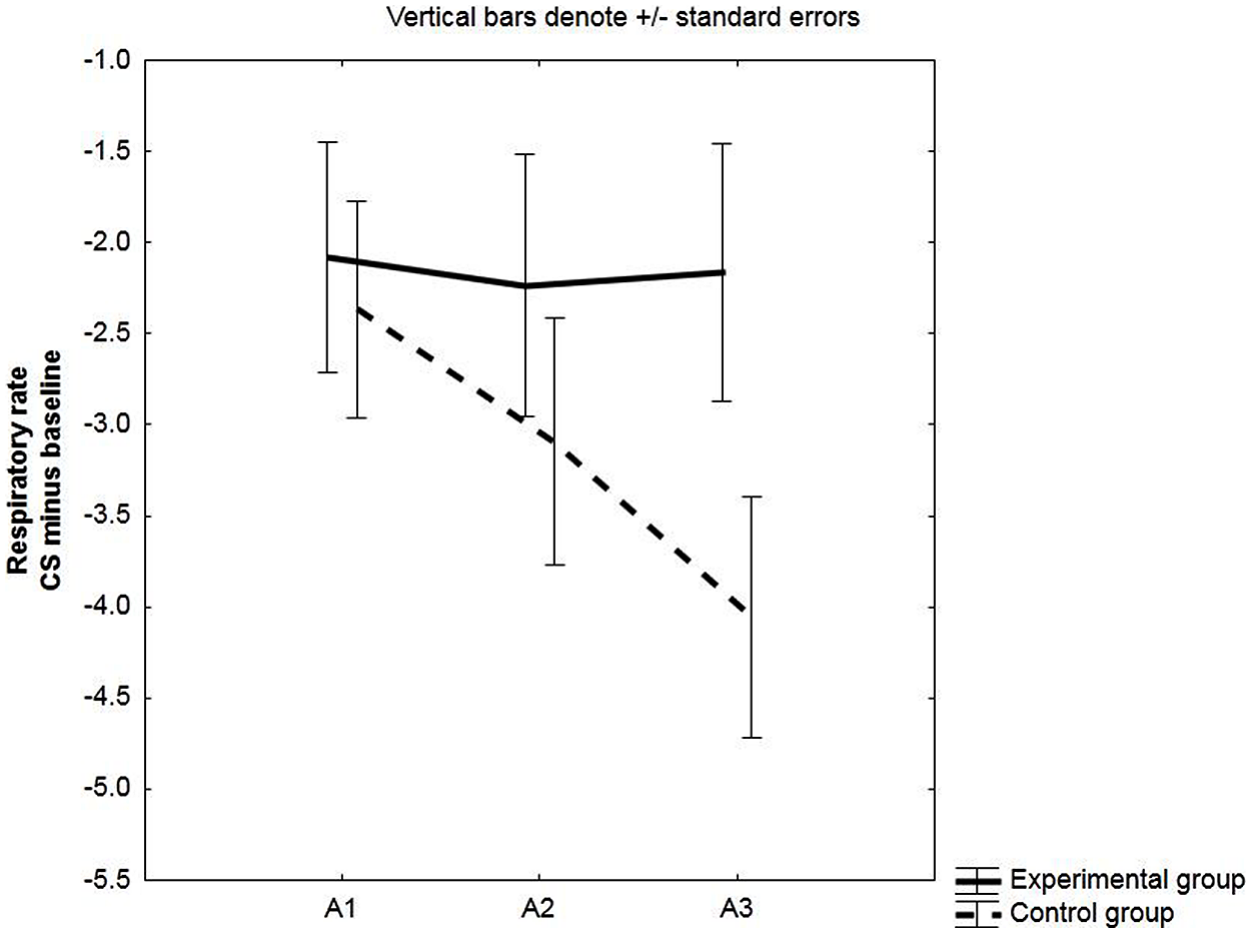
**Change in RR in unaware participants.** Mean changes in RR (in cycles per minute, cpm) during the CS relative to baseline (CS minus baseline) for the experimental and the control group. Respiratory responses were averaged across two acquisition trials (A1, A2, A3).

However, in line with our results in the total group sample, we did observe a linear decrease in RR across acquisition blocks in the control group: *F*(1,32) = 5.83, *p* = 0.02, but not in the experimental group: *F*(1,32) = 0.01, *p* = 0.91, see **Figure 7**. Again, this effect was driven by an increase in RR in the control group during baseline (data not presented).

**FIGURE 8:**
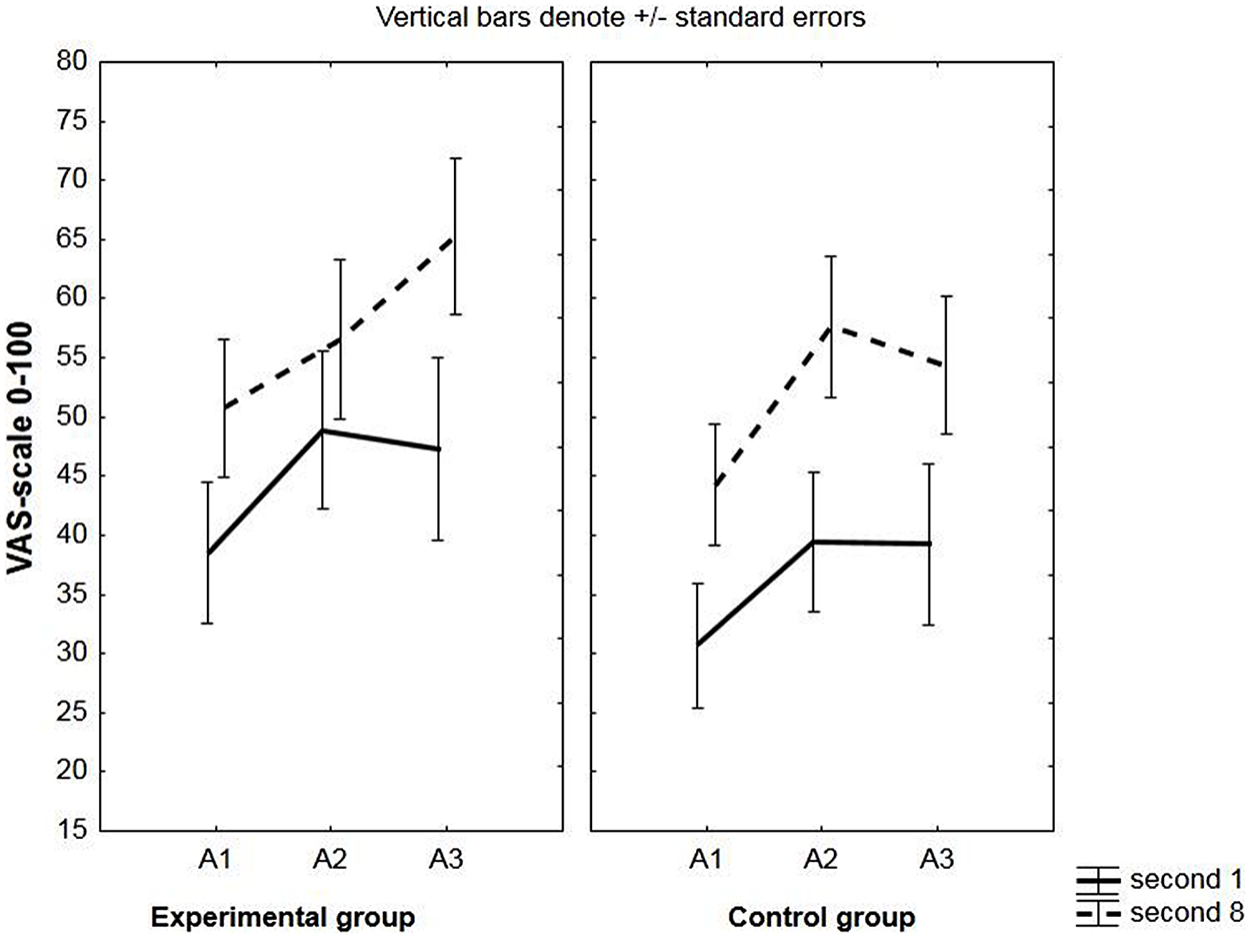
**US-expectancy during the CS-period in the subgroup of unaware participants.** Mean US-expectancy ratings of second 1 and second 8 during the CS on a VAS-scale ranging from 0 (certainly no heavy breathing resistance) to 100 (certainly heavy breathing resistance) for the experimental and the control group during the three acquisition blocks (A1, A2, A3).

#### Tidal Volume (V_T_)

No significant effects involving the group factor were observed in V_T_.

Effect of Group: *F*(1,32) = 0.45, *p* = 0.50, $\eta_{p}^{2}$ = 0.01. Group × Block interaction: *F*(2,64) = 0.9, *p* = 0.41, $\eta_{p}^{2}$ = 0.03.

#### Skin Conductance Response

No significant effects involving the group factor were observed during acquisition: main effect of Group, *F*(1,33) = 1.3, *p* = 0.26, $\eta_{p}^{2}$ = 0.04; Group × Block: *F*(2,66) = 0.46, *p* = 0.63, $\eta_{p}^{2}$ = 0.01.

## Discussion

The aim of the current study was to examine the relationship between interoceptive fear conditioning and CS–US contingency awareness. To this end we first sought to replicate the results of a previous study (Pappens et al., 2013) that suggested fear learning had occurred without declarative knowledge of the CS–US relationship. Second, we aimed to examine fear learning in the subgroup of CS–US unaware participants. Our design consisted of the administration of six explicitly paired presentations of mild breathlessness (CS) followed by strong dyspnea (US) in the experimental group while the control group received the same number of CSs and USs in an explicitly unpaired fashion. Our measures included startle eyeblink EMG, respiratory parameters, skin conductance, a continuous US expectancy measure and a discrete post-experimental CS–US contingency assessment. Based on Pappens et al. (2013), we predicted to observe a dissociation in fear outcomes, that is, fear learning (or: group) effects in startle potentiation and respiration, but not in self-reported US-expectancy. We expected the same pattern to occur in a subgroup of participants who failed to verbalize the CS–US relationship at the end of the experiment.

Our findings confirm that a mild respiratory sensation can acquire fear-evoking properties because of its predictive relationship with an aversive respiratory event. These results corroborate earlier findings demonstrating interoceptive fear conditioning (Acheson et al., 2007, 2012; Pappens et al., 2012, 2014) and they successfully replicate the data of Pappens et al. (2013). Most importantly, we replicated the expected dissociation of the dependent measures: during acquisition experimental and control group differed in fear responding both in startle eyeblink EMG and respiratory measures, but not in US expectancy.

In the second part of this study we examined the relationship between fear learning and declarative knowledge of the CS–US relationship in a more direct way by selecting a subgroup of participants who could not correctly report this relationship despite 100% explicit contingent presentations of CS and US. Despite the absence of propositional knowledge and the lack of fear conditioning effects in US-expectancy, they did display fear learning in startle eyeblink responses and – to a lesser extent – in RR. These results demonstrate fear acquisition without declarative knowledge of the CS–US relationship.

In contrast to Pappens et al. (2013), the current study did include a SCR measurement, but we failed to observe conditioning effects in SCR. Several authors have argued that fear conditioned changes in SCR, just as US-expectancy, may primarily reflect propositional learning (declarative knowledge of the CS–US contingency), whereas startle eyeblink potentiation more directly reflects subcortical emotional learning that can dissociate from propositional learning (e.g., Hamm and Vaitl, 1996; Öhman and Mineka, 2001; Weike et al., 2007; Soeter and Kindt, 2010). However, previous experiments generated mixed results as to whether conditioning of SCR requires ‘conscious’ knowledge of the CS–US relationship (Hamm and Vaitl, 1996; Purkis and Lipp, 2001; Lovibond and Shanks, 2002; Hamm and Weike, 2005; Weike et al., 2007; Sevenster et al., 2014) or whether ‘unconscious’ fear learning also results in conditioned SCRs (Bechara et al., 1995; Knight et al., 2003; Schultz and Helmstetter, 2010). Our results rather support the former hypothesis: we did not observe learning effects in SCR in a group that failed to learn declarative knowledge of the CS–US relationship.

For several reasons, the present interoceptive fear conditioning paradigm seems especially apt for the experimental study of panic. First, 40% of panic attacks are described as coming ‘out of the blue’ (Shulman et al., 1994) although the efficacy of exposure therapy suggests that panic attacks are conditioned events triggered by a cue (McHugh et al., 2009; Meuret et al., 2011). The dissociation observed in our data can offer a nice framework for understanding this apparent paradox. Despite being unable to verbalize the CS–US relationship, participants did display conditioned defensive fear responses. For this reason, our interoceptive fear conditioning paradigm could represent a strong tool for the further study of unexpected panic.

Second, clinically, the dissociation we observed in fear measures could offer an explanation for the high relapse rate observed in anxiety patients after exposure therapy (Vervliet et al., 2013): although verbal assessment indicated their fear had disappeared, residual fear might still have been present at the end of therapy, precipitating the recovery of fear at a later instance. The dissociation observed in our data and the failure of total fear extinction observed in startle eyeblinks in the experimental group (Pappens et al., 2013) shows that our paradigm could serve to study the phenomenon of return of fear.

Third, the fact that we installed fear learning to a fear relevant *respiratory* sensation is of special interest for the study of panic. In the phenomenology of panic, next to cardiac sensations, respiratory symptoms are prevalent (e.g., Ley, 1989; Briggs et al., 1993; Klein, 1993; Kircanski et al., 2009; Meuret et al., 2011) and seem vulnerable for fear conditioning processes. This may be related to the fact that both response systems produce strong internal sensations that may enter awareness and that both are referring to vital functions, the failure of which may represent a sudden life threatening experience causing fear of imminent death. Last, this study represents a successful replication of an earlier experiment (Pappens et al., 2013) corroborating the usefulness of this paradigm as a source of stable and reliable results.

Most studies that reported unconscious fear learning manipulated the level of awareness experimentally by adding a distractor task (e.g., Smith et al., 2005; Tabbert et al., 2006, 2011; Weike et al., 2007), through subliminal conditioning (Öhman and Soares, 1998; Raes et al., 2010) or by obscuring discriminability between the danger and the safety cue (Knight et al., 2003; Schultz and Helmstetter, 2010). Proponents of a single process theory refuted these results based on the lack of a consensus concerning the criteria used to determine the effectiveness of blocking a stimulus from awareness (Sebastiani et al., 2011). A strength of our study is that it created a dissociation without the inclusion of an awareness manipulation. It would, however, be presumptuous to interpret our data as strong evidence for a dual process theory of fear learning since it cannot be excluded that one measure (e.g., the startle eyeblink response) might be more sensitive than another one (e.g., SCR or US expectancy), yet still reflect the same, single learning process. Our data do suggest that different response systems can at least have different activation thresholds in the learning of the contingency between CS and US.

A first limitation of our study is that the effects obtained are rather small. Interoceptive fear conditioning seems overall difficult to install with a homoreflexive paradigm, i.c., a design in which CS and US share the same response system and have similar initial sensory properties (Dworkin, 2000). Future studies should thus focus on the identification of individuals that are more prone to develop fear for harmless interoceptive sensations. For example, we demonstrated that persons scoring high on Fear of Suffocation (a subscale of the Claustrophobia Questionnaire) are more vulnerable for maladaptive breathing during obstructed breathing (Pappens et al., 2013). Also, Melzig et al. (2011) demonstrated that people who score high on Anxiety Sensitivity, are more sensitive to respiratory cues.

Second, since an increase of US expectancy was already present during pre-exposure it is possible that later fear learning effects in US expectancy were masked due to ceiling effects. The increase during pre-exposure is in line with previous results (Pappens et al., 2013) and is likely caused by the inherent relatedness of the mild resistive load (applied as CS) and the breathing occlusion (applied as US). Based on the findings of another study, we have recently argued that pre-existing expectations about the CS–US relationship may indeed hamper detecting the true contingencies (Schroijen et al., 2015).

In summary, this study successfully installed interoceptive fear to mild dyspnea. Fear learning was observed in startle blink EMG and respiratory measures but not in US expectancy and SCR. The same pattern of results was observed in subgroup of participants who failed to acquire declarative knowledge of the CS–US relationship. We propose that our interoceptive fear learning paradigm might serve as an ecologically valid laboratory model for unexpected panic.

## Conflict of Interest Statement

The authors declare that the research was conducted in the absence of any commercial or financial relationships that could be construed as a potential conflict of interest.

## References

[B1] Acheson D. T. Forsyth J. P. Moses E. (2012). Interoceptive fear conditioning and panic disorder: the role of conditioned stimulus–unconditioned stimulus predictability. *Behav. Ther.* 6 174–189. 10.1016/j.beth.2011.06.001 22304889

[B2] Acheson D. T. Forsyth J. P. Prenoveau J. M. Bouton M. E. (2007). Interoceptive fear conditioning as a learning model of panic disorder: an experimental evaluation using 20% CO2-enriched air in a non- clinical sample. *Behav. Res. Ther.* 45 2280–2294. 10.1016/j.brat.2007.04.008 17548049

[B3] Beaver J. D. Mogg K. Bradley B. P. (2005). Emotional conditioning to masked stimuli and modulation of visuospatial attention. *Emotion* 5 67–79. 15755220 10.1037/1528-3542.5.1.67

[B4] Bechara A. Tranel D. Damasio H. Adolphs R. Rockland C. Damasio A. R. (1995). Double dissociation of conditioning and declarative knowledge relative to the amygdala and hippocampus in humans. *Science* 269 1115–1118. 7652558 10.1126/science.7652558

[B5] Blumenthal T. D. Cuthbert B. N. Filion D. L. Hackley S. Lipp O. V. Van Boxtel A. (2005). Committee report: guidelines for human startle eyeblink electromyographic studies. *Psychophysiology* 42 1–15. 10.1111/j.14698986.2005.00271.x 15720576

[B6] Bouton M. E. Mineka S. Barlow D. H. (2001). A modern learning theory perspective on the etiology of panic disorder. *Psychol. Rev.* 108 4–32. 11212632 10.1037/0033-295x.108.1.4

[B7] Briggs A. C. Stretch D. D. Brandon S. (1993). Subtyping of panic disorder by symptom profile. *Br. J. Psychiatry* 163 201–209. 10.1192/bjp.163.2.201 8075912

[B8] Clark R. E. Squire L. R. (1998). Classical conditioning and brain systems: the role of awareness. *Science* 280 77–81. 9525860 10.1126/science.280.5360.77

[B9] De Clerck A. Verschuere B. Crombez G. De Vlieger P. (2006). Psychophysiological analysis (PSPHA): a modular script based program for analyzing psychophysiological data. *Behav. Res. Methods* 38 504–510. 10.3758/BF03192805 17186761

[B10] De Cort K. Griez E. Büchler M. Schruers K. (2012). The role of “interoceptive” fear conditioning in the development of panic disorder. *Behav. Ther.* 1 203–215. 10.1016/j.beth.2011.06.005 22304891

[B11] Dworkin B. R. (2000). “Interoception,” in *Handbook of Psychophysiology*, 2nd Edn, eds Cacioppo J. T. Tassinary L. G. Berntson G. G. (New York: Cambridge University Press), 482–506.

[B12] Hamm A. O. Vaitl D. (1996). Affective learning: awareness and aversion. *Psychophysiology* 33 698–710. 8961792 10.1111/j.1469-8986.1996.tb02366.x

[B13] Hamm A. O. Weike A. I. (2005). The neuropsychology of fear learning and fear regulation. *Int. J. Psychophysiol.* 57 5–14. 10.1016/j.ijpsycho.2005.01.006 15935258

[B14] Johnson P. L. Federici L. M. Shekhar A. (2014). Etiology, triggers and neurochemical circuits associated with unexpected, expected, and laboratory-induced panic attacks. *Neurosci. Biobehav. Rev.* 46 429–454. 10.1016/j.neubiorev.2014.07.027 25130976 PMC4252820

[B15] Kessler R. C. Chiu W. T. Jin R. Ruscio A. M. Shear K. Walters E. E. (2006). The epidemiology of panic attacks, panic disorder, and agoraphobia in the National Comorbidity Survey Replication. *Arch. Gen. Psychiat.* 63 415–424. 16585471 10.1001/archpsyc.63.4.415 PMC1958997

[B16] Kindt M. Soeter M. Vervliet B. (2009). Beyond extinction: erasing human fear responses and preventing the return of fear. *Nat. Neurosci.* 12 256–258. 10.1038/nn.2271 19219038

[B17] Kircanski K. Phil C. Craske M. G. Epstein A. M. Wittchen H.-U. (2009). Subtypes of panic attacks: a critical review of the empirical literature. *Depress. Anxiety* 26 878–887. 10.1002/da.20603 19750553

[B18] Klein D. F. (1993). False suffocation alarms, spontaneous panics, and related conditions: an integrative hypothesis. *Arch. Gen. Psychiatry* 50 306–317. 10.1001/archpsyc.1993.01820160076009 8466392

[B19] Knight D. Nguyen H. Bandettini P. (2003). Expression of conditional fear with and without awareness. *Proc. Natl. Acad. Sci. U.S.A.* 100 15280–15283. 14657356 10.1073/pnas.2535780100 PMC299981

[B20] LeDoux J. E. (1996). *The Emotional Brain.* New York: Simon & Schuster.

[B21] LeDoux J. E. (2000). Emotion circuits in the brain. *Annu. Rev. Neurosci.* 23 155–184. 10.1146/annurev.neuro.23.1.155 10845062

[B22] Ley R. (1989). Dyspneic-fear and catastrophic cognitions in hyperventilatory panic attacks. *Behav. Res. Ther.* 27 549–554. 10.1016/0005-7967(89)90089-2 2684135

[B23] Lovibond P. F. Shanks D. R. (2002). The role of awareness in Pavlovian conditioning: empirical evidence and theoretical implications. *J. Exp. Psychol.* 1 3–26. 11868231

[B24] McHugh R. K. Smits J. A. Otto M. W. (2009). Empirically supported treatments for panic disorder. *Psychiat. Clin. N. Am.* 32 593–610. 10.1016/j.psc.2009.05.005 19716992

[B25] Melzig C. A. Holtz K. Michalowski J. M. Hamm A. O. (2011). Interoceptive threat leads to defensive mobilization in highly anxiety sensitive persons. *Psychophysiology* 48 745–754. 10.1111/j.1469-8986.2010.01150 21073480

[B26] Meuret A. E. Rosenfield D. Wilhelm F. H. Zhou E. Conrad A. Ritz T. (2011). Do unexpected panic attacks occur spontaneously? *Biol. Psychiatry* 70 985–991. 10.1016/j.biopsych.2011.05.027 21783179 PMC3327298

[B27] Mitchell C. J. De Houwer J. Lovibond P. F. (2009). The propositional nature of human associative learning. *Behav. Brain Sci.* 32 183–198. 19386174 10.1017/S0140525X09000855

[B28] Newell B. R. Shanks D. R. (2014). Unconscious influences on decision making: a critical review. *Behav. Brain Sci.* 37 1–19. 10.1017/S0140525X12003214 24461214

[B29] Öhman A. Mineka S. (2001). Fears, phobias, and preparedness: toward an evolved module of fear and fear learning. *Psychol. Rev.* 108 483–522. 11488376 10.1037/0033-295x.108.3.483

[B30] Öhman A. Soares J. J. (1998). Emotional conditioning to masked stimuli: expectancies for aversive outcomes following nonrecognized fear relevant stimuli. *J. Abnorm. Psychol.* 127 69–82. 10.1037//0096-3445.127.1.69 9503652

[B31] Pappens M. Schroijen M. Sütterlin S. Smets E. Van den Bergh O. Thayer J. (2014). Resting heart rate variability predicts safety learning and fear extinction in an interoceptive fear conditioning paradigm. *PLoS ONE* 9:e105054 10.1371/journal.pone.0105054 PMC4152223 25181542

[B32] Pappens M. Smets E. Vansteenwegen D. Van den Bergh O. Van Diest I. (2012). Learning to fear suffocation: a new paradigm for interoceptive fear conditioning. *Psychophysiology* 6 821–828. 22332799 10.1111/j.1469-8986.2012.01357.x

[B33] Pappens M. Van den Bergh O. Vansteenwegen D. Ceunen E. De Peuter S. Van Diest I. (2013). Learning to fear obstructed breathing: comparing interoceptive and exteroceptive cues. *Biol. Psychol.* 92 36–42. 21640786 10.1016/j.biopsycho.2011.05.004

[B34] Purkis H. M. Lipp O. V. (2001). Does affective learning exist in the absence of contingency awareness? *Learn. Motiv.* 32 84–99.

[B35] Raes A. K. Koster E. H. Van Damme S. Fias W. De Raedt R. (2010). Aversive conditioning under conditions of restricted awareness: effects on spatial cueing. *Q. J. Exp. Psychol.* 1 23. 10.1080/17470218.2010.492995 20687009

[B36] Schroijen M. Pappens M. Schruers K. Van den Bergh O. Vervliet B. Van Diest I. (2015). Generalization of fear to respiratory sensations. *Behav. Ther.* (in press) 10.1016/j.beth.2015.004 26459842

[B37] Schultz D. H. Helmstetter F. J. (2010). Classical conditioning of autonomic fear responses is independent of contingency awareness. *J. Exp. Psychol. Anim. Behav. Process.* 36 495–500. 10.1037/a0020263 20973611 PMC6510249

[B38] Sebastiani L. Castellani E. D’Alessandro L. (2011). Emotion processing without awareness: features detection or significance evaluation? *Int. J. Psychophysiol.* 80 150–156. 10.1016/j.ijpsycho.2011.02.019 21392544

[B39] Sevenster D. Beckers T. Kindt M. (2014). Fear conditioning of SCR but not the startle reflex requires conscious discrimination of threat and safety. *Front. Behav. Neurosci.* 8 32 10.3389/fnbeh.2014.00032 PMC3937874 24616672

[B40] Shulman I. D. Cox B. J. Swinson R. P. Kuch K. Reichman J. T. (1994). Precipitating events, locations and reactions associated with initial unexpected panic attacks. *Behav. Res. Ther.* 32 17–20. 8135717 10.1016/0005-7967(94)90079-5

[B41] Smith C. Clark R. Manns J. Squire L. (2005). Acquisition of differential delay eyeblink conditioning is independent of awareness. *Behav. Neurosci.* 119 78–86. 15727514 10.1037/0735-7044.119.1.78 PMC2773180

[B42] Soeter M. Kindt M. (2010). Dissociating response systems: erasing fear from memory. *Neurobiol. Learn. Mem.* 94 30–41. 10.1016/j.nlm.2010.03.004 20381628

[B43] Spruyt A. Clarysse J. Vansteenwegen D. Baeyens F. Hermans D. (2010). Affect 4.0*:* A free software package for implementing psychological and psychophysiological experiments. *Exp. Psychol.* 57 36–45. 10.1027/1618-3169/a000005 20178962

[B44] Tabbert K. Merz C. J. Klucken T. Schweckendiek J. Vaitl D. Wolf O. T. (2011). Influence of contingency awareness on neural, electrodermal and evaluative responses during fear conditioning. *Soc. Cogn. Affect. Neurosci.* 6 495–506. 10.1093/scan/nsq070 20693389 PMC3150860

[B45] Tabbert K. Stark R. Kirsch P. Vaitl D. (2006). Dissociation of neural responses and skin conductance reactions during fear conditioning with and without awareness of stimulus contingencies. *Neuroimage* 32 761–770. 10.1016/j.neuroimage.2006.03.038 16651009

[B46] Vervliet B. Craske M. G. Hermans D. (2013). Fear extinction and relapse: state of the art. *Annu. Rev. Clin. Psychol.* 9 215–248. 10.1146/annurev-clinpsy-050212-185542 23537484

[B47] Vickers K. McNally R. J. (2005). Respiratory symptoms and panic in the National Comorbidity Survey: a test of Klein’s suffocation false alarm theory. *Behav. Res. Ther.* 43 1011–1018. 15967172 10.1016/j.brat.2004.06.019

[B48] Weike A. I. Schupp H. T. Hamm A. O. (2007). Fear acquisition requires awareness in trace but not delay conditioning. *Psychophysiology* 44 170–180. 10.1111/j.1469-8986.2006.00469.x 17241153

